# Intrathecal Injection in a Rat Model: A Potential Route to Deliver Human Wharton’s Jelly-Derived Mesenchymal Stem Cells into the Brain

**DOI:** 10.3390/ijms21041272

**Published:** 2020-02-13

**Authors:** Hyeongseop Kim, Duk L. Na, Na Kyung Lee, A Ran Kim, Seunghoon Lee, Hyemin Jang

**Affiliations:** 1 Department of Health Sciences and Technology, SAIHST, Sungkyunkwan University, 81 Irwon-ro, Gangnam-gu, Seoul 06351, Korea; hyeongseop09@gmail.com (H.K.);; 2 Stem Cell & Regenerative Medicine Institute, Samsung Medical Center, 81 Irwon-ro, Gangnam-gu, Seoul 06351, Korea; 3 Department of Neurology, Samsung Medical Center, Sungkyunkwan University School of Medicine, 81 Irwon-ro, Gangnam-gu, Seoul 06351, Korea; 4 Neuroscience Center, Samsung Medical Center, 81 Irwon-ro, Gangnam-gu, Seoul 06351, Korea; 5 Samsung Alzheimer Research Center, Samsung Medical Center, 81 Irwon-ro, Gangnam-gu, Seoul 06351, Korea; 6 College of Medicine, Sungkyunkwan University, 81 Irwon-ro, Gangnam-gu, Seoul 06351, Korea; 7 Animal Research and Molecular Imaging Center Samsung Biomedical Research Institute, Samsung Medical Center, 81 Irwon-ro, Gangnam-gu, Seoul 06351, Korea; 8 Department of Neurosurgery, Samsung Medical Center, Sungkyunkwan University School of Medicine, 81 Irwon-ro, Gangnam-gu, Seoul 06351, Korea

**Keywords:** intrathecal, mesenchymal stem cell, delivery, migration, injection route

## Abstract

Mesenchymal stem cells (MSCs) are considered as promising therapeutic agents for neurodegenerative disorders because they can reduce underlying pathology and also repair damaged tissues. Regarding the delivery of MSCs into the brain, intravenous and intra-arterial routes may be less feasible than intraparenchymal and intracerebroventricular routes due to the blood–brain barrier. Compared to the intraparenchymal or intracerebroventricular routes, however, the intrathecal route may have advantages: this route can deliver MSCs throughout the entire neuraxis and it is less invasive since brain surgery is not required. The objective of this study was to investigate the distribution of human Wharton’s jelly-derived MSCs (WJ-MSCs) injected via the intrathecal route in a rat model. WJ-MSCs (1 × 10^6^) were intrathecally injected via the L2-3 intervertebral space in 6-week-old Sprague Dawley rats. These rats were then sacrificed at varying time points: 0, 6, and 12 h following injection. At 12 h, a significant number of MSCs were detected in the brain but not in other organs. Furthermore, with a 10-fold higher dose of WJ-MSCs, there was a substantial increase in the number of cells migrating to the brain. These results suggest that the intrathecal route can be a promising route for the performance of stem cell therapy for CNS diseases.

## 1. Introduction

By differentiating into various cell types and inducing regeneration when transplanted into damaged tissue sites, pluripotent stem cells such as embryonic stem cells (ESCs) and induced pluripotent stem cells (iPSCs) hold great promise in stem cell therapy. However, this high pluripotency of ESCs or iPSCs increases the chances of teratoma formation [1]. To evade potential formation of teratomas, various methods such as differentiation into specific lineages, selection of pure progenitor cells, and introduction of suicidal genes or specific substances have been reported [2,3,4,5]. Mesenchymal stem cells (MSCs), on the other hand, are multipotent and when induced are known to differentiate into adipocytes, chondrocytes, and osteoblasts in vitro [6]. From a clinical perspective, this limited potency of MSCs is beneficial in that the chances of teratoma formation are reduced [7,8]. Besides, when injected into the target site, MSCs secrete various paracrine factors that can recover homeostasis and eliminate the cause of the disease [9,10,11,12,13]. 

Optimizing the administration dose and improving the therapeutic efficacy of MSCs must be considered in order to maximize clinical efficacy [14]. Choosing the optimal delivery route is especially important for CNS diseases [15]. The blood–brain barrier (BBB) prevents pathogens that are circulating in the bloodstream from entering the brain [16]. The protective effects of the BBB prevent antibody or chemical drugs from penetrating into the brain parenchyma [17]. Therefore, we expect that MSCs delivered via the intravenous and intra-arterial routes would cross the BBB [18]. However, we previously reported that it is not feasible to penetrate the BBB and that the intraparenchymal and intracerebroventricular routes are more effective for achieving accurate delivery to the brain [11,19,20,21]. 

Compared to these invasive routes, the intrathecal route has several advantages. First, since the cerebrospinal fluid (CSF) flows along the surface of the spinal cord up towards the brain, MSCs can be delivered into the entire neuraxis. One of our previous studies showed that the CSF from normal elderly or Alzheimer patients does not affect the stemness of MSCs [22]. Thus, we expect that the therapeutic potential of the MSCs will not be affected when injected via the intrathecal route. Second, the intrathecal route does not require brain surgery. Therefore, serious complications involving brain surgery such as needle tract injury, infection, and hemorrhage can be avoided. Third, the medical cost and psychological burden associated with surgical procedures will be reduced. Finally, the intrathecal route can be beneficial when applied to neurogenerative disorders such as amyotrophic lateral sclerosis and frontotemporal dementia combined with motor neuron disease, because the route covers not only the brain but also the spinal cord [23,24,25]. Moreover, the safety issues seem to have been resolved, because there were no adverse effects after intrathecal injection of allogeneic bone-marrow-derived mesenchymal stem cells in patients with neurological diseases [26]. The main objective of this study was to examine the biodistribution of MSCs injected intrathecally in a rat model via lumbar puncture. 

## 2. Results

### 2.1. DiD Labeling and Intrathecal Injection of Wharton’s Jelly-Derived MSCs (WJ-MSCs) Were Optimized In Vitro and In Vivo

**Figure 1:**
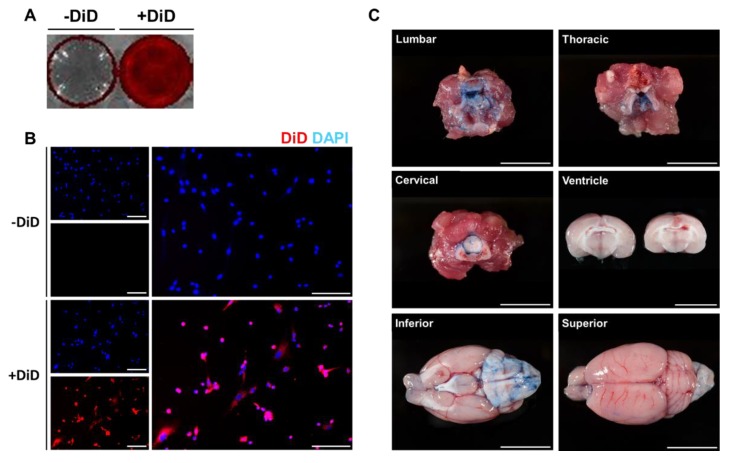
Optimization of DiD labeling and intrathecal delivery. To optimize DiD labeling and intrathecal injection, in vitro and in vivo experiments were carried out, respectively. Fluorescent signals were detected from the DiD-labeled MSCs. (**A**) Red fluorescent signals were visualized from the DiD-labeled MSCs (+DiD) seeded onto 12 well plates. (**B)** The fluorescence of the +DiD cells was also confirmed via fluorescence microscopy where no signals were detected from the -DiD control group. Red: DiD, Blue: DAPI (Scale bar: 500μm). (**C**) To confirm our technique for intrathecal delivery, the trypan blue dye was injected into the lumbar space of rat models (Scale bar: 1 cm).

To detect the migration and distribution of MSCs in the brain and other organs ex vivo, MSCs were labeled with the fluorescent dye DiD before administration. The efficiency of DiD labeling was initially assessed in vitro. DiD-labeled MSCs were seeded onto 12 well plates and images were acquired using an optical imaging instrument (Figure 1A) and fluorescent microscopy (Figure 1B). Compared to the control group (-DiD), high signal intensities were observed from the DiD-MSC group (+DiD). In addition to the optical images, red fluorescent signals were detected around the nucleus only in the +DiD experimental group (Figure 1B). 

Then, intrathecal administration was optimized using a rat model (Figure 1C). Before injecting MSCs via the intrathecal route, trypan blue (1 mL) dye was injected into the L 2-3 intervertebral space of each rat. Rats were sacrificed 15 min after the injection. Based on the autopsy, the lumbar, thoracic, and cervical cord tissues were heavily stained with the trypan blue dye. Through CSF flow, the dye was detected up to the ventral side of the rat brain. According to the coronal sections made of the rat brain, the dye did not penetrate into the brain parenchyma, and the dye was also not detected in the lateral ventricles. 

### 2.2. Intrathecally Delivered WJ-MSCs were Detected in Both the Spinal Cord and the Brain of Rats 12 h after Injection 

**Figure 2:**
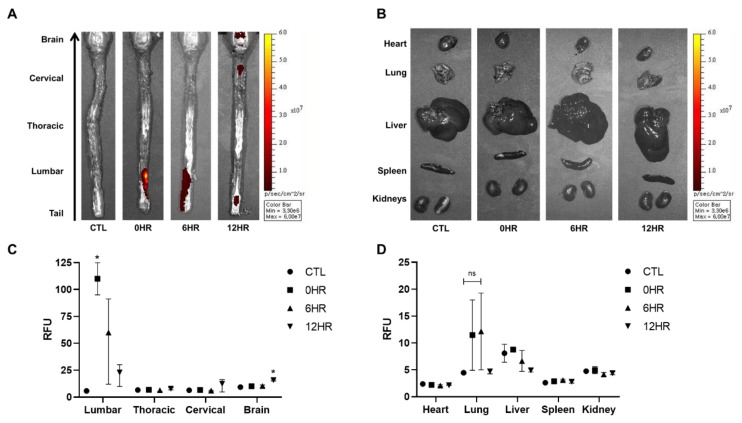
Migration of intrathecally injected Wharton’s jelly-derived mesenchymal stem cells (WJ-MSCs) observed via optical imaging. (**A**,**B**) Ex vivo optical imaging of the brain, spinal cord, and the various organs was conducted at the respective sacrifice time points (0, 6, and 12 h). The red/yellow gradient scale indicates the intensity of the signal. (*n* = 3/group) (**C**,**D**) The signal intensities were quantified using Living Image^®^ 3.1 software. (*n* = 3/group) mean with range. * *p*-value < 0.05 compared to the control group (CTL) of each tissue.

After intrathecal injection of WJ-MSCs, ex vivo optical images were taken to visualize and quantify the distribution and migration of WJ-MSCs (Figure 2). According to the ex vivo optical imaging results, signal intensities were observed from the spinal cord at all time points: 0, 6, and 12 h. Signal intensities, however, were only observed from the brain when sacrificed after 12 h (Figure 2A). Other than the brain, the remaining tissues (heart, lung, liver, spleen, and kidney) showed no signals at any time point (Figure 2B). 

Using optical image analysis software, signal intensities acquired from all images were quantified (Figure 2C,D). The highest signal intensity was observed in the lumbar region of the spinal cord at 0 h when rats were sacrificed right after injection (Figure 2C). The intensity decreased in a timely manner after the 0 h time point. Compared to the thoracic and cervical spinal cords and the brain, the lumbar spinal cord showed the highest signal intensities at 0 and 6 h post-injection. Compared to the 0 h group, a statistically significant increase in signal intensity was observed in the brain at 12 h. Although the highest signal intensities were observed from the lung at 0 and 6 h post-injection, the difference was not significant (Figure 2D).

### 2.3. Migration of Intrathecally Injected WJ-MSCs into the Rat Brain was Confirmed via Quantitative Assessment

**Figure 3:**
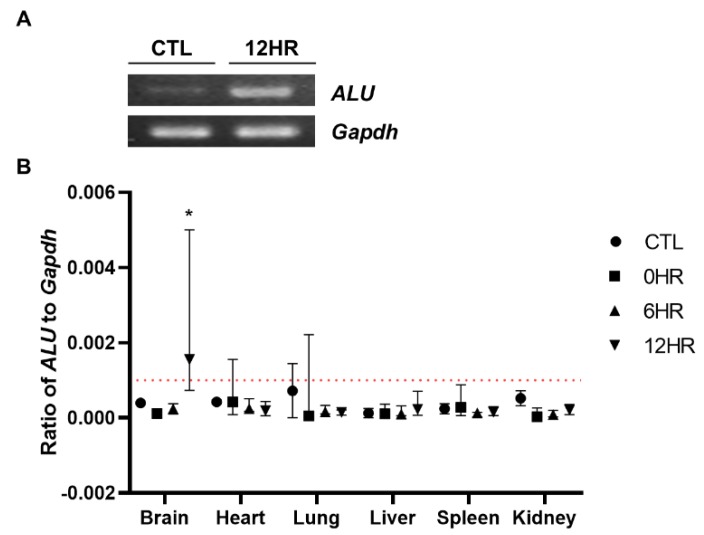
Quantitative assessment of the distribution of intrathecally delivered MSCs. Real-time analysis was performed using the gDNA extracts. (**A**) The human *ALU* sequence was successfully amplified (indicated by a white, intense band) at 12 h post-injection. (**B**) Real-time PCR analysis was performed and the *ALU*/*Gapdh* ratio was calculated. The redline is the limit of detection in this analysis. (*n* = 5/group) mean with range * *p*-value < 0.05 compared to the CTL of each tissue.

To quantify how many intrathecally-injected WJ-MSCs migrated to the rat brain, real-time PCR analysis was performed using the human *ALU* primer. To confirm that the respective genes (human *ALU* and rat *Gapdh*, respectively) were successfully amplified, amplified PCR products were run on a gel using gel electrophoresis. Compared to the control group (CTL, normal SD rat brain where no injections were performed) sample, the *ALU* band was only detected from the sample sacrificed at post 12 h (Figure 3A). Thus, we confirmed that the primers that we have designed correctly amplified the respective target gene. The percentage of residual human MSCs in the rat tissue was calculated by dividing rat *Gapdh* from human *ALU*. In concordance with the ex vivo imaging results, the highest number of MSCs was observed from the group sacrificed at post 12 h in the brain sample (Figure 3B). All of the other samples collected from the varying time points did not reach the limit of the detection threshold (red line). When the ratio was converted to the total number of MSCs, approximately 2.4% of the intrathecally delivered WJ-MSCs were detected in the rat brain. 

### 2.4. Increasing the Cell Injection Dose by Ten-Fold Improves the Migration of Intrathecally Injected WJ-MSCs to the Rat Brain

**Figure 4:**
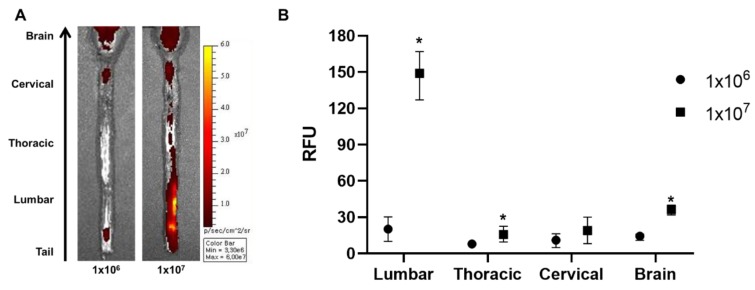
Assessment of the effects of higher injection dose on the intrathecal delivery of WJ-MSCs to the rat brain. An additional injection was performed using a 10-fold higher dose (1 × 10^7^), and the rats were sacrificed after 12 h. (**A**) Ex vivo optical images were taken. (*n* = 3/group) (**B**) Signal intensities were quantified. Mean with range * *p*-value < 0.05 compared to the 1 × 10^6^ group.

The injection dose can contribute towards the successful migration of MSCs to the brain. Therefore, we hypothesized that increasing the injection dose can increase the number of MSCs that will be delivered to the brain. Rats were injected with 1 × 10^7^ (10× the original dose) cells and were sacrificed after 12 h. According to the ex vivo optical imaging results, more positive signals were observed throughout the whole spinal cord when injected with a 10-fold higher dose (Figure 4A). When quantified, compared to the 1 × 10^6^ group, the signal intensity was increased by 7.3-fold for the 1 × 10^7^ group in the lumbar and 2.0-fold in the thoracic region of the spinal cord. The signal intensity was also increased in the brain by 2.6-fold when injected with 1 × 10^7^ cells. Thus, we were able to observe that a greater number of MSCs can be delivered to the brain when the injection dose is increased. 

## 3. Discussion

MSCs have been reported to secrete effective proteins which can cure rare diseases and repair damaged tissues [27]. To successfully achieve the benefits from MSC therapy, various factors such as the injection dose, physiological state of the cells, and administration route must be considered. Especially for CNS diseases such as Alzheimer’s and Parkinson’s disease, the administration route is a crucial factor to consider due to the BBB. Among the various administration routes, we investigated the potential of the intrathecal route as an optimal administration route to deliver MSCs into the rat brain. 

One of the major objectives of this study was to measure how many MSCs can migrate into the rat brain when injected via the intrathecal route. Prior studies have shown that intrathecally (lumbar or cisterna magna) administered proteins or nanoparticles reach the brain parenchyma in the monkey, dog, or mouse [28,29]. However, to the best of our knowledge, our study is the first to examine how many intrathecally injected MSCs migrate towards the brain in an animal model. We expected that if MSCs were injected properly, the CSF flow would allow cells to be distributed widely throughout the brain. As a preliminary experiment, trypan blue dye was injected into the L2-3 intervertebral space of the rats to confirm our technique for intrathecal delivery. The technique introduced in this current study mimics spinal taps performed in human patients. The injected trypan blue successfully stained the spinal cord (lumbar, thoracic, and cervical), the entire ventral side, and the partial dorsal side of the rat brain (Figure 1A).

To quantify the distribution of WJ-MSCs that were injected intrathecally, we used two methods. First, we visualized WJ-MSCs labeled with a fluorescence dye via ex vivo optical imaging. The labeling efficacy was confirmed by in vitro optical and fluorescent microscopic imaging (Figure 1B,C). According to our ex vivo optical imaging analysis, signals were absent at 0 and 6 h post-injection but apparent at 12 h (Figure 2A,C), indicating that it takes between 6 and 12 h for MSCs to migrate up to the brain. Second, we also quantified the migration of WJ-MSCs in different regions of the spinal cord (cervical, thoracic, and lumbar), brain, and other organs by using the human *ALU* primer, which is recommended by the Korean Food and Drug Administration to be used to assess the biodistribution of a stem cell therapy product. It has been reported that the human *ALU* selectively amplifies human-cell-derived-gDNA among non-human samples. Our real-time PCR results were in line with our optical imaging results (Figure 3B). The real-time PCR data showed that the migration of WJ-MSCs into the rat brain was increased significantly at the 12 h time point. On the other hand, transplanted MSCs were not detected in other organs by optical imaging and real-time PCR (Figure 2B,D and Figure 3B). 

Our next question was what percentage of intrathecally-delivered WJ-MSCs would reach the rat brain. According to *ALU* real-time PCR analysis, only 2.4% of injected WJ-MSCs were detected in the rat brain. From these results, we can infer that the majority of the injected MSCs remained in the lumbar region and that a small percentage of cells were able to migrate towards the cervical spinal cord and then up to the brain by 12 h post-injection. Our results have clinical implications in that intrathecal injection may benefit patients who have both cerebral and spinal lesions instead of individuals with isolated cerebral lesions. There have been clinical trials involving patients who harbor both cerebral and spinal lesions, including multiple sclerosis [30,31], amyotrophic lateral sclerosis [23,24,25], spinal cord injury [32], ischemic stroke [33], and ADEM (Acute disseminated encephalitis)-like demyelinating illness [34]. 

There may be several factors that affect the speed or the amount of migration of MSCs from the injection site (lumbar region) up to the brain. First, a previous study from our group showed that the injection concentration can affect the distribution of MSCs after injecting stem cells via the intracerebroventricular route. At higher concentrations, MSCs tended to aggregate, forming clumps, whereas at lower concentrations, MSCs were easily washed out via CSF flow [14]. Second, the cell dose may affect the migration of MSCs towards the brain. When compared to the 1 × 10^6^ cell group, the 1 × 10^7^ injection group showed a 2.6-fold higher migration towards the rat brain (Figure 4). This again underscores the importance of cell dose when performing stem cell therapy and this argument has also been supported by several research papers [35,36]. While we injected MSCs into the spinal cord of a WT rat model, Heejaung Kim and colleagues injected cells into the cisterna magna of a mouse model. When comparing the anatomy between mice and rats, the distance section from the spinal cord (lumbar) to the brain is much longer in comparison to that of mice. Moreover, the total volume of the rat spinal cavity is much greater than that of mice. Compared to that of mice, the CSF volume of rats is approximately 10 times greater [37,38]. Therefore, although the administration route was similar, due to differences in the spinal cavity volume, only about one-tenth of the transplanted cells would have migrated towards the brain in rats. Third, as an alternative to increasing the cell dose, repetitive delivery could also be an option to consider in order to increase the migration of MSCs to the brain [39,40]. Repetitive administration of MSCs can lead to enhanced cell delivery and therapeutic efficacy. However, we chose a single injection of MSCs to evaluate the feasibility of the intrathecal injection route. Fourth, gravity may be another important factor. Unlike rodents, the erect posture of human subjects may interfere with the migration of MSCs toward the brain due to the gravitational pull. Therefore, after administering MSCs intrathecally, keeping patients bed tilted with the head or upper body of the patients placed lower than the lower body may aid in the migration of MSCs up towards the brain. Finally, the homing effect of MSCs may contribute to the migration from the spinal cord toward the brain. Mice or rats with neurodegeneration in the brain would produce more inflammatory mediators and chemoattractants than those of wild type rodents. Therefore, the wild type rats used in our experiment might have contributed to the small percentage of MSC migration to the brain. Furthermore, MSCs may have been strongly attracted to the apparent injuries in the lumbar region which would have affected the migration of the MSCs up towards the rat brain. 

This study has several limitations. First, a rat was chosen over a mouse model because the intervertebral space of mice is too narrow for a fine needle to reach the spinal cavity. While the dye was detected in the ventral regions of the brain, the dye was not observed in the lateral ventricles. This could be due to the rapid flow of CSF from the lateral ventricle to the cerebral aqueduct, or the dye might have been rapidly washed out from the brain. Second, the results of this study cannot be fully translated into human clinical trials because the length of the spinal cord is different between humans and rats. Since the length of the spinal cord is longer in humans, it might take longer than 12 h for MSCs to reach the brain from the injection site. In addition, it has been reported that a few intrathecally injected allogeneic mesenchymal stem cells remained in the cranial sacrum of the horse until 24 h [41]. Third, the migration of MSCs in the rat brain was not followed up after 12 h. From this study, what can be said is that the minimum time required for MSCs to migrate to the brain is around 12 h. Fourth, the therapeutic efficacy of MSCs delivered via the intrathecal route was not evaluated. Although we did not evaluate the therapeutic efficacy of MSCs because the experimental animals we used were healthy, there are many reports in which intrathecally delivered MSCs have shown positive clinical outcomes [42,43].

In summary, we found that MSCs can be delivered to the rat brain when injected via the intrathecal route. At 12 h post-injection, the majority of the injected MSCs were detected in the spinal cord while a small percentage also migrated towards the brain. In addition, the distribution of the human MSCs into other organs other than the spinal cord and brain was not observed. In conclusion, we propose that the intrathecal route has the potential to be used as a route to deliver MSCs into the brain for CNS diseases.

## 4. Materials and Methods 

### 4.1. Ethical Statement

This study was approved by the Institutional Animal Care and Use Committee (IACUC, Approval number: 20170125001, Date: 25 January 2017) of the Samsung Biomedical Research Institute (SBRI) at Samsung Medical Center (SMC). As an accredited facility of the Association for Assessment and Accreditation of Laboratory Animal Care International (AAALAC International), SBRI acts in accordance with the guidelines sets by the Institute of Laboratory Animal Resources (ILAR).

### 4.2. Preparation of Human Wharton’s Jelly-Derived MSCs 

Human Wharton’s jelly-derived mesenchymal stem cells (WJ-MSCs) were grown in culture media comprised of Minimum Essential Medium alpha (MEMα)1× media (Gibco-Invitrogen, Carlsbad, CA, USA), 10% fetal bovine serum (FBS; Biowest, Riverside, MO, USA), and 0.5% gentamicin (Thermo Fisher Scientific, Hudson, NH, USA). Before injection, cells were detached with 0.25% Trypsin-EDTA (Gibco-Invitrogen) and labeled with Vybrant™ DiD cell-labeling solution (Thermo Fisher). After labeling, WJ-MSCs (5 × 10^6^ cells/mL) were suspended in phenol red-free MEMα1× media (Gibco-Invitrogen).

### 4.3. Experimental Animals

Six-week-old Sprague Dawley rats were used for this study (*n* = 35). Rats were purchased from OrientBio Inc. (Gapyeong, Gangwon-do, South Korea,). Thirty-two experimental animals were sacrificed at three post-injection time points, 0 h (*n* = 8), 6 h (*n* = 8), and 12 h (*n* = 8) post 1 × 10^6^ MSC injection, and 12 h (*n* = 3) post 1 × 10^7^ MSC injection. Eight rats were used as a control group. The sacrifice time point was determined by taking into account our knowledge (study in progress) and considering anatomical differences among experimental animals (mice, rats, and other animal models) and humans. They were housed under optimal conditions. Proper food, water, and enrichment were given to the animals, and the cages were refreshed twice per week. At the respective sacrifice time points, the rats were sacrificed using a carbon dioxide (CO_2_) chamber. The following tissues were harvested for analysis: brain, spinal cord (cervical, thoracic, and lumbar), heart, lungs, liver, kidneys, and spleen.

### 4.4. Intrathecal Injection of WJ-MSCs

All the rats were anesthetized with 5% isoflurane, and 2% isoflurane was maintained during the surgical procedure. After shaving and sterilizing the surgical site by povidone-iodine, a skin incision (approximately 3 cm in length) was made over the L2–3 region of the lumbar spinal cord. WJ-MSCs (1 × 10^6^ or 1 × 10^7^ cells suspended in 0.2 mL or 2 mL, respectively) were slowly injected into the L2–3 intervertebral space using a 1 mL syringe. The number of injected MSCs was calculated from previous references including pre-clinical research papers and clinical trials. After the injection, rats were placed upside-down at a 45 °C angle for 15 min to aid the migration of WJ-MSCs toward the brain. After 15 min, the incision site was sutured and sterilized again with povidone-iodine. As a control group (CTL), the same surgical process was performed without WJ-MSC injection.

### 4.5. Ex Vivo DiD Fluorescent Optical Imaging

Using a Xenogen IVIS Spectrum system (Caliper Life Science, Hopkinton, MA, USA), optical images were taken of the organs harvested from the CTL group and rats sacrificed at 0, 6, and 12 h following the intrathecal injection of WJ-MSCs. By referring to a previous study, identical illumination settings (lamp voltage, filters, f/stop, a field of views, and binning) were used when acquiring all images [44]. Fluorescence (emission: 700 nm, excitation: 605 nm for DiD fluorescent dye) was measured as photons per second per centimeter squared per steradian (p/s/cm^2^/sr). Quantification was carried out using Living Image^®^ 3.1 software provided by the optical imaging device manufacturer. Three rectangular ROIs of equivalent sizes were drawn on each spinal cord. The size of each ROI (region of interest) was fixed for each organ and the respective group. The average intensity was measured from each of the ROIs. Statistical analysis was performed using GraphPad Prism 5 (GraphPad Software, CA, USA).

### 4.6. Quantitative PCR

Harvested tissues were stored at -80 °C before DNA extraction. Frozen tissues were ground up using a pre-chilled mortar and pestle. Genomic DNA was extracted using the Gentra Puregene Tissue Kit (QIAGEN, Venlo, Netherlands). Real-time polymerase chain reaction (real-time PCR; Quantstudio 6, Applied Biosystems^TM^ by Life Technologies, MA, USA) was carried out by analyzing 10 ng of genomic DNA per sample using the SYBR Green Master Mix probe (Thermo Fisher Scientific, UK) and primers that targeted the human Arthrobacter luteus (ALU) element. To normalize the samples, rat Gapdh primer was used. The following primers were used for the experiment: 5′-GTC AGG AGA TCG AGA CCA TCC C-3′ (human ALU; forward), 5′-TCC TGC CTC AGC CTC CCA AG-3′ (human ALU; reverse), 5′-TGC CAC TCA GAA GAC TGT GG-3′ (rat Gapdh; forward), and 5′-TTC AGC TCT GGG ATG ACC TT-3′ (rat Gapdh; reverse). A total of 40 cycles were run to amplify the 10 µL PCR reactions (384 well plate) and the steps for each stage were as follows: initial hold (95 °C, 10 min), denaturation (95 °C, 15 sec), annealing (68 °C, 30 sec), and extension (72 °C, 30 sec). The amount of DNA was calculated by fitting the respective threshold cycle (CT) values to a standard curve that was created by varying the number of WJ-MSCs (0, 10^2^, 10^3^, 10^4^, 10^5^, and 10^6^) mixed with rat peripheral blood mononuclear cells (PBMCs) to make a total of 1 × 10^6^ cells. The gDNA concentration and original weight of the harvested tissues were used to calculate the number of residual MSCs in each of the respective tissues. 

### 4.7. Statistical Analysis

All values are represented as mean with range. A Kruskal–Wallis non-parametric test followed by Dunn’s multiple comparison test (Figure 2 and Figure 3) and the Mann–Whitney (one-tailed, Figure 4) non-parametric test were used to assess the significance and a *p*-value ≤ 0.05 was considered statistically significant.
